# Motivations for Adolescent COVID-19 Vaccination: A Comparative Study of Adolescent and Caregiver Perspectives in Germany

**DOI:** 10.3390/children10121890

**Published:** 2023-12-06

**Authors:** Tobias Rothoeft, Folke Brinkmann, Christoph Maier, Dominik Selzer, Christiane Dings, Anna Kuehn, Eva Möhler, Hanna Grote, Alexandra Nonnenmacher, Markus Wenning, Michael Zemlin, Ulf Richter, Thorsten Lehr, Thomas Lücke

**Affiliations:** 1 University Hospital of Pediatrics and Adolescent Medicine, St. Josef-Hospital, Ruhr-University Bochum, 44791 Bochum, Germany; folke.brinkmann@uksh.de (F.B.); christoph.maier@rub.de (C.M.); hanna.grote@posteo.de (H.G.); thomas.luecke@rub.de (T.L.); 2 University Children’s Hospital, 23562 Lübeck, Germany; 3 Airway Research Center North (ARCN), German Center for Lung Research (DZL), 22927 Großhansdorf, Germany; 4 Department of Clinical Pharmacy, Saarland University, 66123 Saarbrücken, Germany; dominik.selzer@uni-saarland.de (D.S.); christiane.dings@uni-saarland.de (C.D.); anna.kuehn@uni-saarland.de (A.K.); thorsten.lehr@mx.uni-saarland.de (T.L.); 5 Department of Child and Adolescent Psychiatry, Saarland University Hospital, 66421 Homburg, Germany; eva.moehler@uks.eu; 6 School of Education and Psychology, Siegen University, 57076 Siegen, Germany; alexandra.nonnenmacher@uni-siegen.de (A.N.); ulf.richter@zv.uni-siegen.de (U.R.); 7 Medical Association, Westfalen-Lippe, 48151 Münster, Germany; markus.wenning@aekwl.de; 8 Department of General Pediatrics and Neonatology, Saarland University Hospital, 66421 Homburg, Germany; michael.zemlin@uks.eu

**Keywords:** vaccine hesitancy, adolescents, COVID-19, public health

## Abstract

Given the crucial role of vaccination in halting the COVID-19 pandemic, it is imperative to understand the factors that motivate adolescents to get vaccinated. We surveyed adolescents and their accompanying guardians scheduled to receive a COVID-19 vaccination (Comirnaty) in an urban region in Germany in mid-2021 regarding their motivation for getting vaccinated and collected data on their sociodemographic characteristics, medical history, vaccination status, and any history of COVID-19 infection in the family. We also queried information strategies related to the SARS-CoV-2 pandemic. Motivations for getting vaccinated were similar among adolescents and their parents. The primary reasons for vaccination were protection against SARS-CoV-2-related illness and gaining access to leisure facilities. This was not influenced by gender, health status, migration background, or the presence of chronic or acute diseases. The percentage of parents who had received SARS-CoV-2 immunization and the proportion of parents with a high level of education were higher among study participants than in the general population. Adolescents were especially willing to be vaccinated if they came from a better educational environment and had a high vaccination rate in the family. Emphasizing the importance of vaccination among all segments of the population and removing barriers to vaccines may lead to an ameliorated acceptance of COVID-19 vaccines.

## 1. Introduction

Children and adolescents represent a minority of patients with severe COVID-19 disease courses, as they are mostly asymptomatic or show minor symptoms such as fever, cough, or a sore throat. Few will suffer severe complications of the infection such as pediatric inflammatory multiorgan syndrome (PIMS), the main pediatric complication of SARS-CoV-2 infection, or post-COVID syndrome (PCS). The mortality of hospitalized pediatric patients is described as 0.1–0.18% [1,2]. The elderly and those with underlying health conditions are more severely affected [3]. However, adolescents are a population with potential for high transmission. This highlights the need for vaccination to help protect more vulnerable populations [4,5].

Dozens of vaccine development programs have been initiated in response to the emergence of SARS-CoV-2, resulting in the development of safe and effective COVID-19 vaccines. In Germany, COVID-19 vaccines have been available since January 2021, and since August 2021, mRNA-based COVID-19 vaccines have been recommended by the German Standing Committee on Vaccination (STIKO) for the immunization of adolescents aged 12–17 years.

However, vaccine hesitancy is a significant barrier to achieving broad vaccination coverage against COVID-19. Generally, vaccine hesitancy is a global issue and has been rated among the top ten threats worldwide [6,7]. A survey conducted in Europe in 2020 showed that almost 20% of respondents were unsure about receiving a vaccine for COVID-19, and a further 7.2% indicated that they did not want to get vaccinated [8]. Understanding vaccine acceptance [9], hesitancy, and resistance [10,11] to COVID-19 vaccinations is therefore of paramount importance to ensure the rapid and requisite uptake of an eventual vaccine. Several factors can contribute to vaccine hesitancy, including complacency, inconvenience, lack of confidence in vaccines, and costs [12]. Few studies have so far directly queried adolescents and their caregivers on their attitudes towards and perceptions of COVID-19 vaccinations [13,14,15]. This study aims to add knowledge about factors motivating an adolescent population and their caregivers to actively pursue obtaining a COVID-19 vaccination for children and adolescents directly after the vaccination was officially recommended.

## 2. Methods

### 2.1. Background and Study Design

The current observational study was conducted as part of a pilot project carried out in the Siegen-Wittgenstein region in Germany between 30 July and 30 September 2021. The present sample was an ad hoc sample. The University of Siegen, Saarland University (Department of Clinical Pharmacy), University Children’s Hospital Bochum, Saarland University Hospital Clinic for General Pediatrics and Neonatology, and Saarland University Hospital Clinic for Child and Adolescent Psychiatry, Psychosomatics, and Psychotherapy conducted the study, with the support of the Siegen vaccination center.

### 2.2. Participant Recruitment and Eligibility

Adolescents aged 12 to 17 years and their caregivers were offered a SARS-CoV-2 vaccination (BNT162b2 by Biotech/Pfizer) prior to the official recommendation for vaccination. The European Medicines Agency’s human medicines committee (CHMP) had already approved the use of BNT162b2 by Biotech/Pfizer before the start of our study. During the initial three weeks of the study phase, vaccination for adolescents aged 12 to 17 years was not recommended by the STIKO, but this recommendation was changed on August 18th. The vaccination campaign was announced in local newspapers, on the radio, and on the University of Siegen homepage. The inclusion criteria for the study were as follows: (a) being a participating adolescent or the parent of a participating adolescent, (b) the adolescent being minimum 12 years of age, (c) the adolescent attending a secondary school or vocational school in the Siegen district or having matriculated at the University of Siegen, (d) the adolescent receiving the COVID-19 vaccination, and (e) voluntary participation in the survey. Exclusion criteria were based on contraindications to the COVID-19 vaccination, such as known hypersensitivity to any vaccine ingredient (for all contraindications, see the Robert Koch Institute (RKI) information leaflet on vaccination against COVID-19 [16]). Recruitment for and education about the study took place at the Siegen vaccination center. Participants had to be volunteers, and they did not receive any incentive to complete the survey. Adolescents and their parents were asked if they wanted to participate in the study survey after receiving the vaccination. If they agreed, consent forms were handed out and written consent was obtained by the study physician. Adolescents aged 16 years or older who presented without parents were also informed about the study with a specific information sheet and then, if capable of understanding the purpose of the study, gave consent to the study physician, according to the General Data Protection Regulation (GDPR) Articles 8 and 9. Informed consent was provided in multiple languages.

### 2.3. Survey Instrumentation and Administration

Participants completed the study questionnaires online during the medically determined waiting period after the vaccination, using their mobile devices or a tablet provided by the study leaders. Access to the online questionnaire was provided via a QR code or web address. The questionnaire answered by the parents or legal guardians included questions on sociodemographic backgrounds of the parent/guardian and adolescent (including gender, essential worker status, place of residence, educational and first-generation migration background), chronic illness status of the adolescent about to be vaccinated, vaccination status, and history of COVID-19 infection of the family. Information strategies regarding the SARS-CoV-2 pandemic and motivation for the vaccination of the adolescents according to the parents were also queried. These responses were based on a Likert-type scale with available responses of “totally agree” (1), “rather agree” (2), “partly” (3), “rather disagree” (4), or “totally disagree” (5).

This study also utilized a questionnaire that was completed by the adolescents, which consisted of questions pertaining to their sociodemographic characteristics and migration background, their health status, and their reasons for getting vaccinated. The questions related to the last two items were based on a Likert-type scale. The survey was provided in multiple languages. Moreover, information regarding problematic internet use as well as the mental health and quality of life of adolescents were surveyed. For this, results were published elsewhere [17,18].

### 2.4. Sample Size Determination

In this study, the sample consisted of ad hoc participants recruited during the operational hours of the Siegen vaccination center. Given the emergent context and the need for timely data collection, the study did not utilize a formal power analysis to calculate a predetermined sample size. Instead, the sample size was contingent on the number of eligible and consenting individuals during the study period, with the goal of including as wide a participant base as possible within the constraints of the setting and timeframe.

### 2.5. Data Analysis

Data processing, analysis, and generation of figures were accomplished using the R programming language version 4.1.3 [19]. Data analysis was conducted using ordered logistic regression to evaluate the Likert scale responses, controlling for covariates and comparing groups with the MASS package in R. Continuous variables were analyzed with t-tests, and categorical variables were assessed using Fisher’s exact test, if appropriate. Statistical significance was established at *p*-values less than 0.05.

### 2.6. Ethical Considerations

The study was conducted in compliance with the Declaration of Helsinki and was approved by the Ethics Committee of the Medical Association of Westphalia-Lippe and Westphalian Wilhelms University (file number: 021-372-f-S).

## 3. Results

From the initial 1477 survey completions by adolescent participants, 51 were excluded as they were 18 years or older, and 13 were excluded due to missing age information. Additionally, 31 adolescent participants completed the survey multiple times, leading to the exclusion of 34 survey completions from the analyzed dataset. The survey completions that provided the most information were kept. One completed survey was removed from the adolescent dataset due to 100% missing survey answers. This resulted in a final sample of 1378 unique adolescent survey completions, which accounted for 93.3% of the initial sample. Of the respondents, 681 (49.4%) were female, 655 (47.5%) were male, and 13 (0.9%) identified as diverse.

**Figure 1:**
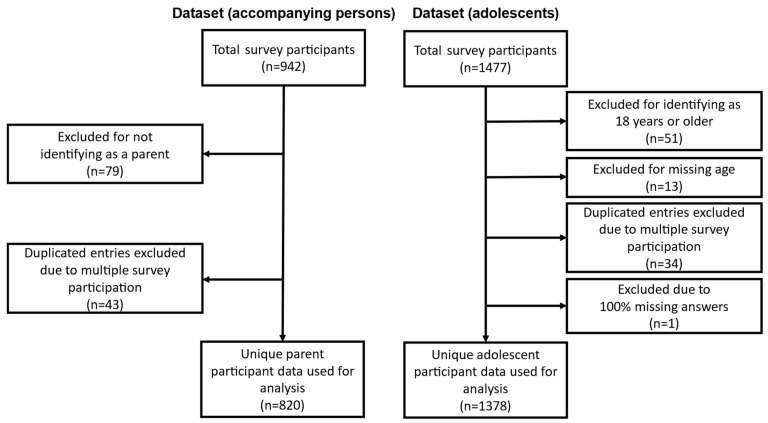
Data selection: surveys completed by accompanying persons and adolescents.

A total of 942 accompanying persons completed the survey, out of whom 863 identified themselves as parents of participating adolescents and were included in the study. Among them, 41 participants completed the survey multiple times, resulting in the exclusion of 43 survey completions from the analyzed dataset. The survey completions that provided the most information (i.e., had the highest number of answered questions and completeness of provided sociodemographic data) were retained, resulting in a final sample of 820 unique participating parent survey completions, which accounted for 87.0% of the original sample. Figure 1 provides a detailed summary of the data selection process for the study participant surveys. Fisher’s exact test revealed significant differences in the gender distribution (“diverse” gender excluded) between participants with and without first-generation migration background (*p* = 0.02). The majority of participants, 95.5% (1316), were born in Germany, while 61 (4.4%) were born in other countries. Of the participants, 83.3% (1148) did not have a first-generation migration background, while 16.7% (230) did have a first-generation migration background (see Table 1).

The mean age of the adolescents was 14.3 years (SD: 1.6). Participants without a first-generation migration background had a mean age of 14.2 years (SD: 1.6), while those with a first-generation migration background had a mean age of 15.0 years (SD: 1.7). The application of a two-sided t-test revealed significant differences in age between these two groups (*p* < 0.001). Among all participants, 81 (5.9%) reported having a chronic disease, with asthma being the most commonly mentioned condition (34.6%). Of those with a first-generation migration background, eight (3.5%) reported having a chronic disease, while seventy-three (6.4%) of those without a first-generation migration background reported having a chronic disease. Overall, 96.6% of participants reported their health status as “good” or “very good or excellent”, while only 2.6% described their health status as “below average” or “poor”.

**Figure 2:**
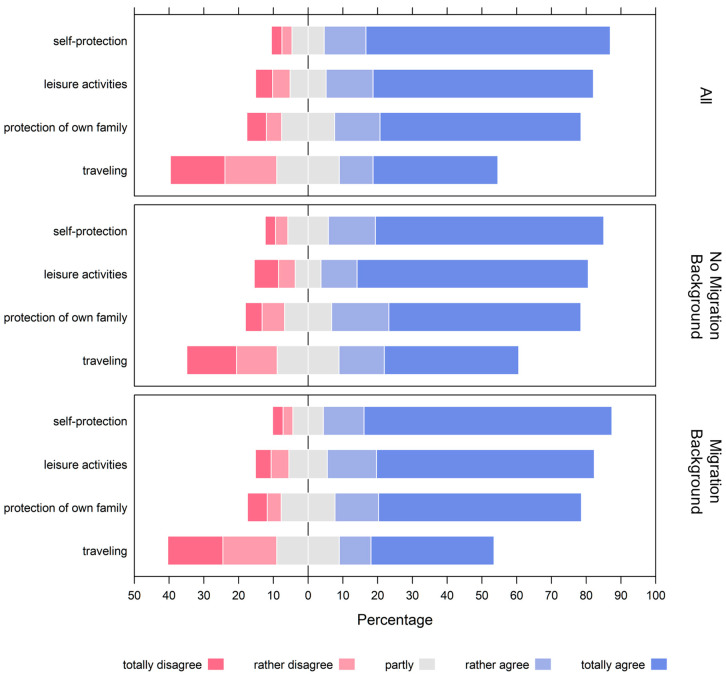
Reasons for adolescents receiving a COVID-19 vaccination, as answered by all adolescents (n = 1378), those without first-generation migration background (n = 1148), and those with a first-generation migration background (n = 230). For numerical data, the reader is kindly referred to Supplementary Table S1.

The primary motivations for vaccination among adolescents were concern about contracting COVID-19 (82.3%), access to leisure facilities (76.9%), and protecting family members (70.9%). The ability to travel was less important to adolescent participants, with only 45.6% citing it as motivation (see Figure 2 and Supplementary Table S1).

Using ordered logistic regression and controlling for migration background, gender, age, and health status, we examined the association between migration background and reasons for COVID-19 vaccination. For this, the health status variable was dichotomized as “poor” (combining “below average” and “poor”) and “good” (combining “good” and “very good or excellent”). Our analysis revealed that migration background was not significantly associated with differences in any reason for vaccination (*p* ≥ 0.05). However, age was significantly associated with reasons for COVID-19 vaccination. Specifically, each additional year of age was associated with a lower likelihood of prioritizing self-protection as a reason for vaccination (OR 0.89; 95% CI 0.82–0.96) and a higher likelihood of prioritizing the ability to travel (OR 1.08; 95% CI 1.02–1.15) and having access to leisure facilities (OR 1.08; 95% CI 1.01–1.16) as reasons for vaccination.

The present study also analyzed survey data from 820 accompanying parents. Of these, 624 (76.1%) identified themselves as mothers of the adolescent, while 196 identified as fathers (23.9%). Of the participating parents, 654 (79.8%) were born in Germany, 57 (7.0%) were not born in Germany, and 109 (13.3%) did not provide an answer to this question. Education level was also assessed, with high-level education indicating completion of high school/grammar school, medium-level education indicating general secondary school, and low-level education indicating education up to primary school/basic secondary school. In 45.9% of participating households, the highest level of education was a university degree or a high school diploma, in 28.7% it was a medium level of education, and in 7.9% it was a low level of education. The level of education was not stated by 17.6% of participants. In households with a migration background, a university degree or a high school diploma was the highest level of education attained in 43.9% of cases. If the migration background was unknown, 98.2% of participants did not provide information about their education level. Among these participants, with a mean age of 45.5 years, 616 (75.1%) identified as female and 200 (24.4%) identified as male. When examining participants with respect to their first-generation migration background, the mean age was 45.8 years for those without a first-generation migration background, 43.9 years for those with a first-generation migration background, and 44.4 years for those with unknown migration backgrounds. A one-way ANOVA revealed a statistically significant difference in mean age between the three groups (*p* = 0.02). When examining gender differences across these groups, 75.7% of those without a first-generation migration background identified as female and 23.9% as male. Among those with a first-generation migration background, 75.4% identified as female and 24.6% identified as male. Among participants with unknown migration backgrounds, 71.6% identified as female and 27.5% identified as male. Here, Fisher’s exact test could not reveal significant differences in gender distribution between these three groups (*p* = 0.58). For a summary of the sociodemographic data, see Table 2.

In this study, we found that a high percentage of interviewed parents had already received a COVID-19 vaccination: at the start of the study, the general population had vaccination rates of approximately 62% in the age group of 18–59 years and approximately 87% in the group of 60 years and older (see Supplementary Figure S1). However, in this study, in 91.8% of cases (see Table 2), at least one parent (90.6% of mothers and 85.5% of fathers) had been vaccinated, despite 98.4% of parents being younger than 60 years old. The vaccination rate of mothers without a migration background was significantly higher, at 92.8%, when compared to mothers with a migration background, who had a vaccination rate of 73.7% (Fisher’s exact test *p*-value < 0.001). Similarly, fathers without a migration background had a higher vaccination rate, of 87.2%, compared to fathers with a migration background, who had a vaccination rate of 70.2% (Fisher’s exact test *p*-value 0.001). Furthermore, we observed that 45.0% (369) of siblings in the interviewed families had already been immunized against COVID-19. Among families with migration backgrounds, 31.6% of siblings had been vaccinated, while 46.2% of siblings in families without migration backgrounds had been vaccinated (Fisher’s exact test *p*-value 0.004). A summary of sociodemographic information and the vaccination and infection status of the parent group can be found in Table 2.

Regarding parents’ sources of information on SARS-CoV-2, we found that the majority obtained information from the internet (53.2%, answer items “definitely yes” or “probably yes”). Family doctors (26.3%), social media (24.4%), family members (17.9%), and school or university (11.5%) were less frequently reported as sources of information. Other media sources were reported by 35.5% of parents but were not specified further.

Using ordered logistic regression, it could be shown that there were significant differences (*p* = 0.003) in information retrieval about SARS-CoV-2 using the internet between parents with first-generation migration background and parents without a migration background, after controlling for age, gender, and education level of the participants. The odds ratio (OR) for parents without a migration background was 0.51 (95% CI 0.27–0.92), indicating that they were less likely to obtain information about SARS-CoV-2 using the internet compared to parents with a migration background. Migration background was not a significant predictor (*p* ≥ 0.05) for any other type of information retrieval about SARS-CoV-2. In terms of gender, male parents were less likely to obtain information from school/university (“definitely no” or “probably no”) compared to females (OR 0.51; 95% CI 0.34–0.76). Education level was also found to be a significant predictor of information retrieval. Parents with a lower or medium education background were less likely to gather information from other media (OR 0.46; 95% CI 0.24–0.88 and OR 0.64; 95% CI 0.45–0.91, respectively) and more likely to retrieve information from social media sources (OR 2.30; 95% CI 1.37–3.88 and OR 2.10; 95% CI 1.53–2.88, respectively) compared to parents with higher education. Age was associated with a lower likelihood of obtaining SARS-CoV-2-related information from family members (per year of age OR 0.97; 95% CI 0.95–1.00). Furthermore, parents who did not provide information about their migration background predominantly (98.2%) did not answer these questions.

**Figure 3:**
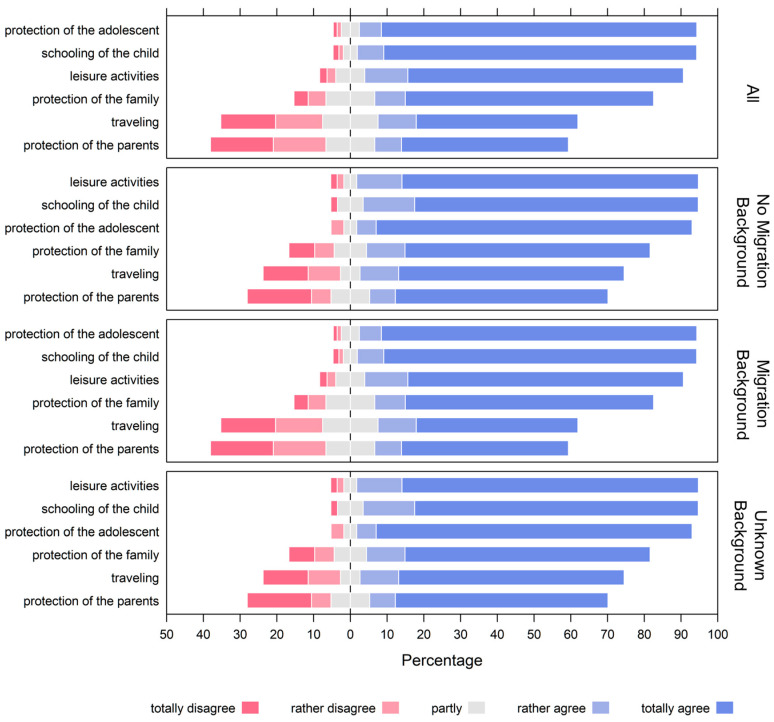
Reasons for adolescents receiving a COVID-19 vaccination, as answered by all parents (n = 820), parents without first-generation migration background (n = 654), those with a first-generation migration background (n = 57), and those with an unknown migration background (n = 109). For numerical data, the reader is kindly referred to Supplementary Table S2.

The survey showed that the protection of the adolescent and the schooling of the adolescents were the most important reasons for parents to vaccinate their children against SARS-CoV-2, with 91.8% and 92.4% of parents, respectively, indicating that they “totally agree” or “rather agree” with these reasons. The ability of adolescents to take part in leisure activities was also an important motivation for vaccination for 86.7% of parents, while the ability to travel was important to 54.4% of parents. Protection of parents and other family members was mentioned by 52.6% and 75.8% of parents, respectively (see Figure 3 and Supplementary Table S2).

Ordered logistic regression was used to investigate the association between the reasons for vaccinating adolescents against SARS-CoV-2 and demographic and health-related factors, including migration background, gender of the parent, previous SARS-CoV-2 infection in the family, age, and chronic illness of the adolescent. Migration background was not significantly associated with any reason for vaccinating adolescents against SARS-CoV-2 (*p* ≥ 0.05). However, the age of the child was associated with a lower importance placed on protecting the adolescent (per year of age OR 0.82; 95% CI 0.72–0.94) and a lower likelihood of prioritizing in-person schooling (per year of age OR 0.86; 95% CI 0.76–0.99). Chronic illness of the adolescent was associated with a lower likelihood of prioritizing the ability to travel (OR 0.59; 95% CI 0.37–0.96), the ability to take part in leisure activities (OR 0.53; 95% CI 0.31–0.92), and in-person schooling (OR 0.42; 95% CI 0.23–0.83). However, chronic illness was not a primary driver for parents choosing to vaccinate their adolescents to protect them (OR 0.82; 95% CI 0.72–0.94). Interestingly, a slightly smaller fraction of parents of chronically ill adolescents (86.7%) chose to vaccinate their adolescents to protect them (“totally agree” or “rather agree”) compared to parents of adolescents without chronic illnesses (92.5%). Moreover, our analysis revealed that in all cases (protection of the family, protection of the adolescents, ability to travel, and participation in leisure activity), parents were more likely to prioritize these reasons for vaccination than adolescents. For the protection of the family, parents were 1.41 times (41%) more likely to agree with its importance, as indicated by an OR of 1.41 with a 95% CI of 1.16–1.69. In terms of the protection of the adolescent, the likelihood of parents agreeing was higher by a factor of 2.54 (154% more), with an OR of 2.54 and a 95% CI of 2.02–3.22. Regarding the ability to travel, parents showed a 28% higher likelihood of agreement, reflected in an OR of 1.28 and a 95% CI of 1.09–1.51. Lastly, for participation in leisure activities, parents were 72% more likely to agree, as shown by an OR of 1.72 and a 95% CI of 1.42–2.09.

Specifically, the odds of parents agreeing with the importance of these reasons were higher than those of adolescents (OR 1.41; 95% CI 1.16–1.69 for the protection of the family, OR 2.54; 95% CI 2.02–3.22 for the protection of the adolescent, OR 1.28; 95% CI 1.09–1.51 for the ability to travel, and OR 1.72; 95% CI 1.42–2.09 for participation in leisure activities).

## 4. Discussion

Motivations for vaccination were similar between caregivers and adolescents and focused on protecting themselves against infection as well as enabling participation in leisure activities. Parents also expressed a strong incentive for vaccination to allow for the in-person schooling of their adolescents. Overall, parents attributed greater importance to all queried reasons for vaccination than adolescents did.

To analyze the conducted study in the context of local and nationwide incidence and vaccination rates, incidence data were fetched from the RKI (https://survstat.rki.de, accessed on 24 October 2023) via a workflow derived from Dings and coworkers [5] and vaccination rates were gathered from the RKI vaccination repository (https://github.com/robert-koch-institut/COVID-19-Impfungen_in_Deutschland, accessed on 24 October 2023).

**Figure 4:**
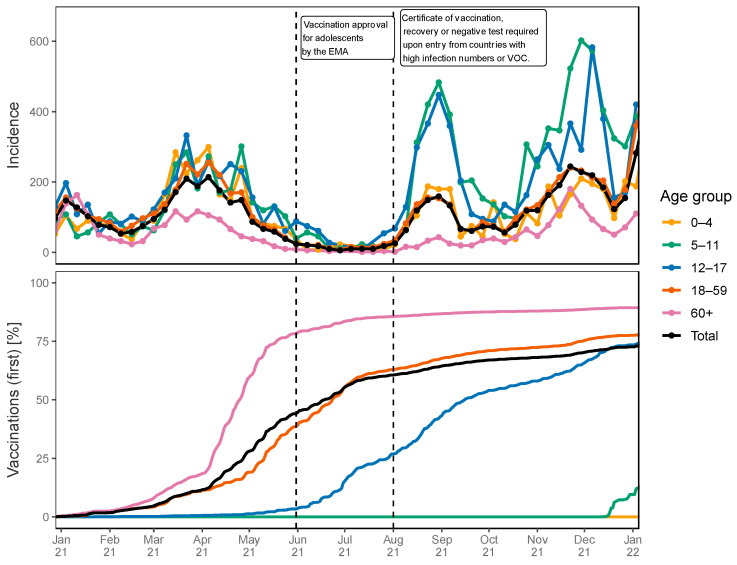
Seven-day incidence rate per 100,000 inhabitants (**upper** panel) and the rates of first vaccinations (**lower** panel) for different age groups over time in the Siegen-Wittgenstein study region stratified by age group. School holidays are marked in blue, the study time frame is marked in red. Dashed lines indicate the date of approval of SARS-CoV-2 vaccinations for adolescents by the EMA and the resolution of a law stating that individuals entering Germany from countries with high infection numbers or VOC require a certificate of vaccination, recent recovery from COVID-19, or a negative test result.

For this, 7-day incidence rates and rates of first vaccinations were plotted over time for different age groups (0–4, 5–11, 12–17, 18–59, and 60+) as depicted in Figure 4. The period of the conducted study is highlighted with a red panel.

During the study period, the incidence rate among adolescents aged 12–17 years (depicted via the blue line) peaked more than twice as high as the population average. The peak incidence rate was close to 500 and hence approximately 0.5% of adolescents had a PCR-confirmed infection with SARS-CoV-2 within a week (see upper panel of Figure 4). The start of the study (30 July 2021) coincided with a time when about 25% of the population of the Siegen-Wittgenstein region had received their first vaccination (see lower panel of Figure 4). However, it should be noted that the number of vaccinations was reported based on the location of the vaccination, not the place of residence of the vaccinated individual, and hence, the fraction of vaccinated inhabitants might be biased.

Approximately 21 days after the start of the study, and during the official school holiday period, a certificate of vaccination, recorded recovery from a previous infection, or negative PCR test was required to enter Germany after travelling to high-incidence countries.

To compare local pandemic and vaccination metrics with the surrounding vicinity and Germany, Supplementary Figure S1 presents the incidence and vaccine rates over time for the entire federal state North Rhine-Westphalia (of which the study region, Siegen-Wittgenstein, is a part) and for Germany. The fractions of vaccinated adolescents were very similar at the beginning of the study (25.3%, 27.7%, and 25.1% in Siegen-Wittgenstein, North Rhine-Westphalia, and Germany, respectively). However, the weekly incidence rate in adolescents was higher in Siegen-Wittgenstein during the study period in comparison to North Rhine-Westphalia and Germany.

In the described parent cohort, over 93% had at least one family member who was already vaccinated. At the time of the study, 62% of individuals in the general population in the age group of 18–59 years had received at least one vaccine dose. Therefore, vaccinated parents were overrepresented in our sample compared to the general population. However, the parents and siblings of adolescents were significantly less likely to be vaccinated if the parents stated a first-generation migration background. These differences in family vaccination coverage hold even if controlling for the age of the participating parent via generalized linear modelling (*p* < 0.001). These findings are in line with findings from a review by Mipatrini and coworkers [20].

Of the accompanying parents, 45.9% stated a high level of education for themselves and 39.1% for their partners. In 56.1% of participating households, at least one individual had a high level of education. This group is overrepresented in our study (*p* < 0.001) compared to the general population, where only about 33.5% of German citizens have a high level of education [21]. Interestingly, parents with higher education levels are described as more likely to worry about vaccine safety [22]. Other studies show correlations between low education levels and vaccine refusal [23], a notion that is supported by the formation of the study population. Consistent with some previous studies, education and income were related to willingness to receive a vaccine [11]. Despite the general preference for obtaining information about SARS-CoV-2 from the internet (54.2%), a lower education level showed a strong association with the preference for gathering information about SARS-CoV-2 from social media if controlling for first-generation migration background, age, and gender. In the studied cohort, the likelihood of first-generation migrants using the internet for information retrieval was lower than for parents without a migration background. Moreover, male parents were less likely to gather information about SARS-CoV-2 from school or university. This might be due to the still unequal care arrangement among parents and the closer contact of mothers to the school system of their children that persisted throughout the pandemic [24].

In the presented parent survey, protection of the adolescents, in-person schooling, and the ability to participate in leisure activities were the most important reasons for the SARS-CoV-2 vaccination of their children. Here, a first-generation migration background, the gender of the parent, and previous SARS-CoV-2 infections in the family were not significant predictors for a change in preferences in any reason for vaccination. For adolescents of lower ages, parents generally put a higher emphasis on the importance of the protection of their children than parents of older adolescents. Travelling, in-person schooling, as well as participating in leisure activities were less important for participants that identified themselves as parents of children affected by chronic illness.

In the study area, the percentage of secondary school pupils with non-German citizenship was between 10 and 12% [25]. However, in the analyzed cohort, only 4.4% of adolescents scheduled to receive a vaccination were not born in Germany, indicating that adolescents with a migration background were slightly underrepresented in this study. This finding is consistent with previous studies conducted in Europe and North America [26,27,28]. A potential explanation for this underrepresentation may be a language barrier, as the study was announced in local newspapers and on the radio. Additionally, families with migration backgrounds may seek out alternative sources of information regarding vaccinations. According to the responsible government department (“Ministerium für Kinder, Familie, Flüchtlinge und Integration des Landes Nordrhein-Westfalen”), 22% of non-German pupils living in the study area are from Syria, 11.7% from Romania, 7.4% from Iraq, 4.8% from Turkey, and 3.9% from Russia. However, in the analyzed cohort, 18.8% were from Syria, 1.2% from Romania, none had been born in Iraq or Turkey, and 10.9% were from Russia (18.8% originating from countries belonging to the former USSR). Therefore, individuals of Romanian and Iraqi descent were underrepresented in the study population, while adolescents from Russia were overrepresented. Adolescents of Syrian descent participated roughly in the expected proportion. The proportion of adolescents who were 16 years or older was higher in the group with a first-generation migration background compared to the group without a first-generation migration background. However, it is unclear from the data collected whether this difference was at least partially due to the fact that adolescents who were 16 years or older did not require parental consent for vaccination.

These findings raise questions about the generalizability of the results of studies conducted on COVID-19 vaccine acceptance in other countries. In a study conducted in 2020, the question “If a COVID-19 vaccine is proven safe and effective and is available, I will take it” showed widely differing vaccine acceptance rates in different countries. Acceptance rates varied from nearly 90% in China to less than 55% in Russia [29], suggesting that concerns or misconceptions specific to certain communities, as well as religious or philosophical beliefs, may interfere with vaccination programs. These concerns may not be universally applicable, as Russian-born adolescents appeared at the vaccination center in greater numbers than expected.

For the surveyed adolescents, self-protection, access to leisure facilities, as well as protection of the family were important reasons for vaccination. Here, first-generation migration background, health status, and gender were not significant predictors of changing preferences in any reason to get vaccinated. Older adolescents put a higher emphasis on the ability to travel and having access to leisure facilities in comparison to younger participants. As in the parent survey, older age was significantly associated with a lower emphasis on the protection of the adolescent. Protection against COVID-19, as expected and reported by other studies, was one of the main reasons [14,30], as perceived risks are key factors for positive vaccination decisions. In particular, the protection of other family members or close contacts was not the main reason to get vaccinated, but this may be less surprising given the fact that a high percentage of the parents were already vaccinated, so that indirect protection in this cohort may not have been necessary. Interestingly, the motivation of the adolescents was not modified by health status, migration background, being of poor health, or having a chronic illness [31]. Whether gender differences in the willingness to get vaccinated exist seems to be a matter of debate [10,11,23] but our results support the notion that overall willingness to receive a COVID-19 vaccine is not influenced by gender.

The most frequently mentioned diagnosed medical condition was asthma, whereas only one adolescent suffered from a malignant disease. Of the adolescents having asthma, 93% described themselves as being in good health. Although 5.8% of participants described themselves as suffering from a chronic illness, these diseases are not necessarily to be regarded as severe. The only factor changing the emphasis on positive factors for getting vaccinated was a family history of COVID-19 infections. The experience of being in quarantine for 14 days probably brings a greater emphasis on social participation and less emphasis on self-protection.

It is noteworthy that vaccine hesitancy towards COVID-19 vaccines does not seem to impact the routine vaccination schedule. In the studied region, over 97% of children attending primary school have received the measles–mumps–rubella (MMR) vaccine [32,33]. Therefore, current vaccine hesitancy or resistance appears to be limited to COVID-19 vaccines and is not reflective of general vaccine hesitancy. This finding aligns with previous reports that have identified vaccine hesitancy as a phenomenon that is both “vaccine-specific” and “varying over time and place” [34].

In our study, we acknowledge several limitations that may impact the interpretation and generalizability of our findings. Notably, the absence of a formal power analysis means that our sample size was not calculated to detect a specific effect size with predetermined statistical power. This methodological choice was due to the exploratory nature of the study and the necessity of rapid data collection in response to changing public health recommendations. Therefore, the findings should be interpreted with caution, particularly when considering the representativeness and generalizability of the results to other populations. Given that the study was conducted at a vaccination center, the observed cohort had already decided to receive a vaccination. Additionally, the study was initiated before the German Standing Committee on Vaccination (STIKO) had recommended COVID-19 vaccination for adolescents, although the European Medicines Agency’s human medicines committee (CHMP) had already approved extending the indication for the COVID-19 vaccine Comirnaty (BNT162b2 by Biotech/Pfizer, Mainz, Germany) to include use in children aged 12 to 15 years. Moreover, our study lacks comprehensive information on the majority of adolescents who had not received vaccinations at the time of data collection. These missing data limit our ability to draw definitive conclusions about the factors influencing vaccination uptake among this demographic and may introduce potential biases in our results. There was a substantial proportion of participants from the parent group (n = 109) who did not provide information regarding their migration background. This group also exhibited a low participation rate in answering questions about their education level and sources of information about COVID-19. It is plausible that language barriers may have contributed to this lack of response, which could have inadvertently excluded or underrepresented the perspectives of individuals from diverse linguistic backgrounds. Lastly, our study does not include data on adolescents who received vaccinations but did not complete the survey. It is possible that families with language barriers or other accessibility issues may have been excluded from our sample. This exclusion could result in an underestimation of the true vaccination uptake among certain subgroups.

In general, a high level of education of either the person getting vaccinated or the caregiver seems to be the best predictor of vaccination [35]. Several studies conducted on the acceptance of COVID-19 vaccines hinted at lower acceptance rates for the vaccination among children and adolescents in families and milieus of low income, less education, and if the parents themselves were not vaccinated [36,37,38,39]. Our data show that the population appearing at our vaccination center in active pursuit of a COVID-19 vaccination came from a higher-educated environment and had a higher vaccination rate in the family [13] and is less likely to have a migration background than the general population. Other predicting factors and especially reasons for vaccine hesitancy and resistance may only be used with caution. As described in other studies, these motivations can change over time [29,40] as the perception of vaccine risks or perceptions of increasing or decreasing disease threats evolve [41]. They are also under the continuous influence of a multiplicity of other surrounding factors [42].

## 5. Conclusions

Vaccine hesitancy is a complex, multifaceted issue, manifesting differently across distinct population segments, particularly among individuals with lower educational levels. Our study underscores the importance of developing tailored public health strategies to effectively navigate these perceived barriers to vaccination. To maximize vaccine uptake, it is crucial to create clear and accessible health communication strategies that resonate with these specific communities. By addressing these disparities, an environment could be fostered that supports informed decision-making and promotes wider acceptance of vaccination. Future research should focus on possible interventions to attain these goals.

## Figures and Tables

**Table 1:** Demographic data of the adolescents stratified by migration background.

			First-Generation Migration Background	
		All	Without	With
n		1378	1148	230
Gender	missing	29 (2.1%)	25 (2.2%)	4 (1.7%)
	female	681 (49.4%)	551 (48.0%)	130 (56.5%)
	male	655 (47.5%)	562 (49.0%)	93 (40.4%)
	diverse	13 (0.9%)	10 (0.9%)	3 (1.3%)
Age	mean (±SD)	14.3 (1.6)	14.2 (1.6)	15.0 (1.7)
	12–13 years	480 (34.8%)	427 (37.2%)	53 (23.0%)
	14–15 years	515 (37.4%)	444 (38.7%)	71 (30.9%)
	16–17 years	383 (27.8%)	277 (24.1%)	106 (46.1%)
Chronic disease	missing	4 (0.3%)	4 (0.3%)	0 (0.0%)
	no	1293 (93.8%)	1071 (93.3%)	222 (96.5%)
	yes	81 (5.9%)	73 (6.4%)	8 (3.5%)
General health	missing	11 (0.8%)	9 (0.8%)	2 (0.9%)
	poor	6 (0.4%)	6 (0.5%)	0 (0.0%)
	below average	30 (2.2%)	24 (2.1%)	6 (2.6%)
	good	345 (25.0%)	272 (23.7%)	73 (31.7%)
	very good or excellent	986 (71.6%)	837 (72.9%)	149 (64.8%)

**Table 2:** Sociodemographic data of the parents stratified by migration background.

			First-Generation Migration Background		
		All	Without	With	Unknown
n		820	654	57	109
Gender	missing	3 (0.4%)	3 (0.5%)	0 (0.0%)	0 (0.0%)
	female	616 (75.1%)	495 (75.7%)	43 (75.4%)	78 (71.6%)
	male	200 (24.4%)	156 (23.9%)	14 (24.6%)	30 (27.5%)
Age	missing	3 (0.4%)	2 (0.3%)	0 (0.0%)	1 (0.9%)
	mean (±SD)	45.5 (6.5)	45.8 (6.3)	43.9 (5.5)	44.4 (8.2)
	<60 years	807 (98.4%)	644 (98.5%)	56 (98.2%)	107 (98.2%)
	≥60 years	10 (1.2%)	8 (1.2%)	1 (1.8%)	1 (0.9%)
Mother and/or father vaccinated	missing	17 (2.1%)	12 (1.8%)	2 (3.5%)	3 (2.8%)
	yes	753 (91.8%)	613 (93.7%)	44 (77.2%)	96 (88.1%)
	no	50 (6.1%)	29 (4.4%)	11 (19.3%)	10 (9.2%)
SARS-CoV-2 infection of accompanying parent	missing	1 (0.1%)	0 (0.0%)	0 (0.0%)	1 (0.9%)
	yes	24 (2.9%)	18 (2.8%)	3 (5.3%)	3 (2.8%)
	no	795 (97.0%)	636 (97.2%)	54 (94.7%)	105 (96.3%)
SARS-CoV-2 infection of other family members	missing	17 (2.1%)	3 (0.5%)	0 (0.0%)	14 (12.8%)
	yes	93 (11.3%)	66 (10.1%)	12 (21.1%)	15 (13.8%)
	no	710 (86.6%)	585 (89.4%)	45 (78.9%)	80 (73.4%)
Education level	missing	144 (17.6%)	28 (4.3%)	9 (15.8%)	107 (98.2%)
	low	65 (7.9%)	55 (8.4%)	10 (17.5%)	0 (0.0%)
	medium	235 (28.7%)	221 (33.8%)	13 (22.8%)	1 (0.9%)
	high	376 (45.9%)	350 (53.5%)	25 (43.9%)	1 (0.9%)

## Data Availability

The data presented in this study are available on request from the corresponding author. The data are not publicly available due to restrictions apply to the availability of these data, which were used under license for the current study.

## Supplementary Materials

The following supporting information can be downloaded at: https://www.mdpi.com/article/10.3390/children10121890/s1, Figure S1: Seven-day SARS-CoV-2 incidence per 100,000 inhabitants and the fraction of vaccinated individuals (first vaccine dose) in Germany, federal state of North Rhine-Westphalia, and the Siegen-Wittgenstein study region stratified by age group. School holidays are marked in blue, the study time frame is marked in red. Dashed lines indicate the date of approval of SARS-CoV-2 vaccinations for adolescents by the EMA and the resolution of a law stating that individuals entering Germany from countries with high infection numbers or VOC require a certificate of vaccination, recent recovery from COVID-19, or a negative test result.; Table S1: Reasons for receiving a COVID-19 vaccination as answered by the adolescents; Table S2: Reasons for adolescents receiving a COVID-19 vaccination as answered by the parents, stratified by migration background.
