# Supplementary material for: Motivations for Adolescent COVID-19 Vaccination: A Comparative Study of Adolescent and Caregiver Perspectives in Germany
Source: Children (Basel). 2023 Dec 6;10(12):1890. doi: 10.3390/children10121890 (PMC10742286; doi:10.3390/children10121890)
Supplement: Supplementary file 1 [file children-10-01890-s001.zip › children-2714949-supplementary.pdf]

Supplementary Information

Motivations for Adolescent COVID-19 Vaccination: A Comparative Study of Adolescent and Caregiver Perspectives in Germany

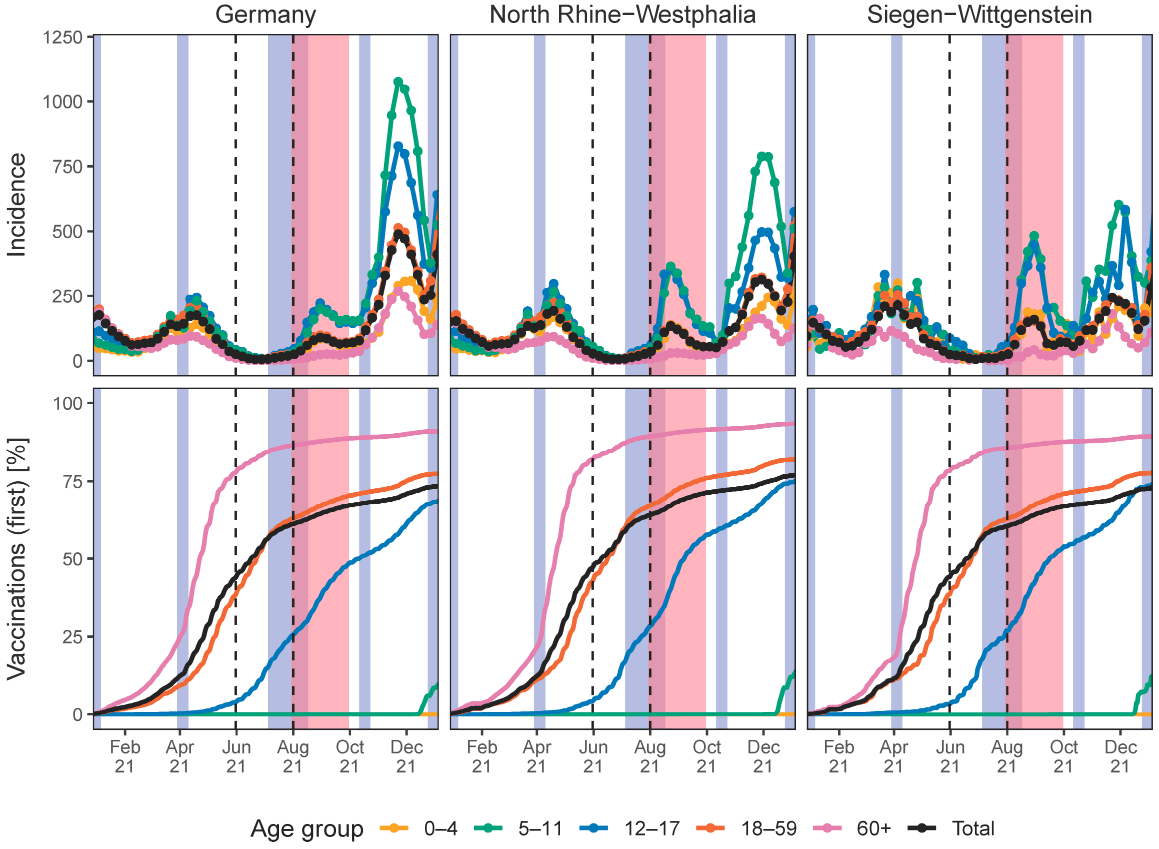

Figure S1. Seven-day SARS-CoV-2 incidence per 100,000 inhabitants and the fraction of vaccinated individuals (first vaccine dose) in Germany, federal state of North Rhine-Westphalia, and the Siegen-Wittgenstein study region stratified by age group. School holidays are marked in blue, the study time frame is marked in red. Dashed lines indicate the date of approval of SARS-CoV-2 vaccinations for adolescents by the EMA and the resolution of a law stating that individuals entering Germany from countries with high infection numbers or VOC require a certificate of vaccination, recent recovery from COVID-19, or a negative test result.

Table S1. Reasons for receiving a COVID-19 vaccination as answered by the adolescents.

		First generation migration background		
**Reason for vaccination**		**all**	**without**	**with**
	Total (n)	1378	1148	230
Protection of own family	missing	53 (3.8%)	45 (3.9%)	8 (3.5%)
	totally agree	797 (57.8%)	670 (58.4%)	127 (55.2%)
	rather agree	181 (13.1%)	143 (12.5%)	38 (16.5%)
	partly	209 (15.2%)	178 (15.5%)	31 (13.5%)
	rather disagree	61 (4.4%)	46 (4.0%)	15 (6.5%)
	totally disagree	77 (5.6%)	66 (5.7%)	11 (4.8%)
Self-protection	missing	34 (2.5%)	28 (2.4%)	6 (2.6%)
	totally agree	969 (70.3%)	818 (71.3%)	151 (65.7%)
	rather agree	165 (12.0%)	134 (11.7%)	31 (13.5%)
	partly	128 (9.3%)	101 (8.8%)	27 (11.7%)

	rather disagree	40 (2.9%)	32 (2.8%)	8 (3.5%)
	totally disagree	42 (3.0%)	35 (3.0%)	7 (3.0%)
Travelling	missing	80 (5.8%)	70 (6.1%)	10 (4.3%)
	totally agree	495 (35.9%)	406 (35.4%)	89 (38.7%)
	rather agree	134 (9.7%)	104 (9.1%)	30 (13.0%)
	partly	248 (18.0%)	207 (18.0%)	41 (17.8%)
	rather disagree	205 (14.9%)	178 (15.5%)	27 (11.7%)
	totally disagree	216 (15.7%)	183 (15.9%)	33 (14.3%)
Leisure activities	missing	38 (2.8%)	29 (2.5%)	9 (3.9%)
	totally agree	873 (63.4%)	720 (62.7%)	153 (66.5%)
	rather agree	186 (13.5%)	162 (14.1%)	24 (10.4%)
	partly	144 (10.4%)	127 (11.1%)	17 (7.4%)
	rather disagree	69 (5.0%)	58 (5.1%)	11 (4.8%)
	totally disagree	68 (4.9%)	52 (4.5%)	16 (7.0%)

Table S2. Reasons for adolescents receiving a COVID-19 vaccination as answered by the parents, stratified by migration background.

			First-generation migration background		
Reason for vaccination		**all**	**without**	**with**	**unknown**
	Total (n)	820	654	57	109
Protection of the adolescent	missing	9 (1.1%)	6 (0.9%)	1 (1.8%)	2 (1.8%)
	totally agree	704 (85.9%)	561 (85.8%)	49 (86.0%)	94 (86.2%)
	rather agree	49 (6.0%)	39 (6.0%)	3 (5.3%)	7 (6.4%)
	partly	40 (4.9%)	36 (5.5%)	2 (3.5%)	2 (1.8%)
	rather disagree	9 (1.1%)	6 (0.9%)	2 (3.5%)	1 (0.9%)
	totally disagree	9 (1.1%)	6 (0.9%)	0 (0.0%)	3 (2.8%)
Protection of the family	missing	18 (2.2%)	15 (2.3%)	1 (1.8%)	2 (1.8%)
	totally agree	554 (67.6%)	437 (66.8%)	38 (66.7%)	79 (72.5%)
	rather agree	68 (8.3%)	56 (8.6%)	6 (10.5%)	6 (5.5%)
	partly	109 (13.3%)	90 (13.8%)	5 (8.8%)	14 (12.8%)
	rather disagree	39 (4.8%)	31 (4.7%)	3 (5.3%)	5 (4.6%)
	totally disagree	32 (3.9%)	25 (3.8%)	4 (7.0%)	3 (2.8%)
Protection of the parents	missing	22 (2.7%)	18 (2.8%)	1 (1.8%)	3 (2.8%)
	totally agree	372 (45.4%)	287 (43.9%)	33 (57.9%)	52 (47.7%)
	rather agree	60 (7.3%)	51 (7.8%)	4 (7.0%)	5 (4.6%)
	partly	109 (13.3%)	88 (13.5%)	6 (10.5%)	15 (13.8%)
	rather disagree	117 (14.3%)	96 (14.7%)	3 (5.3%)	18 (16.5%)
	totally disagree	140 (17.1%)	114 (17.4%)	10 (17.5%)	16 (14.7%)
Travelling	missing	23 (2.8%)	20 (3.1%)	1 (1.8%)	2 (1.8%)
	totally agree	361 (44.0%)	274 (41.9%)	35 (61.4%)	52 (47.7%)
	rather agree	85 (10.4%)	69 (10.6%)	6 (10.5%)	10 (9.2%)
	partly	124 (15.1%)	107 (16.4%)	3 (5.3%)	14 (12.8%)
	rather disagree	105 (12.8%)	90 (13.8%)	5 (8.8%)	10 (9.2%)
	totally disagree	122 (14.9%)	94 (14.4%)	7 (12.3%)	21 (19.3%)
Leisure activities	missing	8 (1.0%)	6 (0.9%)	0 (0.0%)	2 (1.8%)
	totally agree	615 (75.0%)	488 (74.6%)	46 (80.7%)	81 (74.3%)
	rather agree	96 (11.7%)	78 (11.9%)	7 (12.3%)	11 (10.1%)
	partly	65 (7.9%)	53 (8.1%)	2 (3.5%)	10 (9.2%)
	rather disagree	20 (2.4%)	17 (2.6%)	1 (1.8%)	2 (1.8%)

	totally disagree	16 (2.0%)	12 (1.8%)	1 (1.8%)	3 (2.8%)
Schooling of the child	missing	8 (1.0%)	6 (0.9%)	0 (0.0%)	2 (1.8%)
	totally agree	699 (85.2%)	560 (85.6%)	44 (77.2%)	95 (87.2%)
	rather agree	59 (7.2%)	45 (6.9%)	8 (14.0%)	6 (5.5%)
	partly	31 (3.8%)	24 (3.7%)	4 (7.0%)	3 (2.8%)
	rather disagree	10 (1.2%)	9 (1.4%)	0 (0.0%)	1 (0.9%)
	totally disagree	13 (1.6%)	10 (1.5%)	1 (1.8%)	2 (1.8%)
